# Identification of the *AKCDPK* gene family and *AkCDPK15* functional analysis under drought and salt stress

**DOI:** 10.1371/journal.pone.0325453

**Published:** 2025-06-11

**Authors:** Penghua Gao, Ying Zou, Min Yang, Lifang Li, Ying Qi, Jianwei Guo, Yongteng Zhao, Jiani Liu, Jianrong Zhao, Feiyan Huang, Lei Yu

**Affiliations:** 1 College of Agronomy, Yunnan Urban Agricultural Engineering and Technological Research Center, Yunnan Provincial Science and Technology Department, Kunming University, Kunming, China; Assam Agricultural University Faculty of Agriculture, INDIA

## Abstract

Konjac is one of the important economic crops for poverty alleviation in mountainous areas of Yunnan Province, China. However, there are always various biotic and abiotic stress during its growth, leading to production reduction and quality decline. Calcium-dependent protein kinases (CDPKs) are an important class of genes involved in calcium ion signal transmission within plant tissue cells, yet their presence and functions in konjac remain unexplored. This study aimed to identify the members of the *AkCDPK* gene family in the *Amorphophallus konjac* genome and understand their evolution and responses to various stresses. A total of 29 *AkCDPK* genes were identified and categorized into four subgroups that unevenly distributed across 12 chromosomes. Most *AkCDPK* have undergone purifying selection during evolution. Cis-acting element analysis revealed that several *AkCDPK* are involved in phytohormone induction, defence, stress response, and plant development. Expression analysis indicated tissue specificity, and responses to salt, drought, and *Pectobacterium carotovorum* subsp*. carotovorum* stress. *AkCDPK15,* encoding 582 amino acids, was cloned. *AkCDPK15* was mainly localised on the cell membrane, and overexpression in tobacco revealed that it can positively regulate the tolerance of transgenic tobacco strains to salt and drought stress. These findings provide a theoretical foundation for future research on the function of the *CDPK* gene family in *A. konjac*, potentially aiding in the development of stress-resistant konjac varieties.

## Background

The complex growth environment of plants means that they may be subjected to various biotic and abiotic stresses during their lifetimes. Therefore, plants adapt to various harsh environments by regulating their internal environment. These defence responses include a burst of reactive oxygen species (ROS), Ca^2+^-mediated signalling pathways, and mitogen-activated protein kinase cascade reactions, etc. [1–3]. Calcium-dependent protein kinases (CDPKs) can direct Ca^2+^ signals to phosphorylation pathways, serving as a sensor and responder to Ca^2+^ when plants are under stress [4–7].

CDPKs are serine/threonine protein kinases widely distributed in plants [8]. The proteins encoded by *CDPK* usually contain four domains: calcium-binding (CBD), N-terminal variable (VNTD), Ser/Thr protein kinase (PKD), and self-inhibitory junction (JD) [9]. The CBD contains EF-hand-shaped domains that bind Ca^2+^. Among the four domains, the PKD is highly conserved and contains Ser/Thr phosphorylation sites, which are involved in activating kinase activity. The VNTD region of most *CDPK*-encoded proteins contain myristoyl or palmitoyl sites; the JD, usually located near the PKD, inhibits kinase activity and prevents the occurrence of regulatory pathways by binding to pseudo substrates [7,10]. When plants perceive unfavourable environmental conditions, specific Ca^2+^ signals are generated within the cells. Ca^2+^ directly binds to the EF-hand structure, relieves the self-inhibitory effect of kinases, realises kinase substrate phosphorylation, and activates kinase activity. In addition, the PKD can bind adenosine triphosphate (ATP) or guanosine triphosphate (GTP) and transfer γ-phosphate groups to receptor hydroxyl residues, activate substrates, and induce various physiological reactions in plants [11,12].

Recent studies have indicated that *CDPKs* play a role in plant growth and stress response. When rice (*Oryza sativa*) is subjected to water deficiency, *OsCDPK5* enhances its resistance to drought by increasing its photosynthetic activity [13]. Potato (*Solanum tuberosum*) *CDPKs*, such as *StCDPK28*, have similar functions in response to water deficiency [14]. *OsCPK12* positively regulates rice productivity by prolonging its growth period [15]. *SpCPK33* enhances cold resistance in tomatoes by reducing ROS accumulation and regulating the expression of stress-related genes [16]. In wheat (*Triticum aestivum*), *TaCDPK27* negatively regulates wheat resistance to powdery mildew (*Blumeria graminis* f. sp. *tritici*) by participating in the regulation of ROS production, antioxidant enzyme activity, and programmed cell death processes [17]. And the positive regulatory gene *TaCDPK7* in wheat is involved in tolerance to *Puccinia striiformis* f. sp. *tritici* by regulating the content of hydrogen peroxide and the expression of related defence genes [18]*.*

Konjac is an economically important crop in China. *Amorphophallus konjac* is the most-commonly cultivated konjac species in agriculture. However, *A. konjac* is often threatened by drought, salt stress, and soft rot during cultivation, which hinders the development of the konjac industry. As the *CDPK* gene family acts as receptors for calcium ions, playing important roles in regulating plant growth and development, hormone responses, and a variety of biotic and abiotic stresses responses, we suspected that it plays a key role in the water deficit, salt, and disease resistance of *A. konjac*. At present, the complete genome sequencing of *A. konjac* has laid the foundation for exploring the expression, evolution, and functional characteristics of *CDPK* in *A. konjac* at the whole genome level [19,20]. Consequently, to explore the function and genetic evolution of *AkCDPK*, bioinformatics were used to identify and analyse the *AkCDPK* gene family members from the whole genome of *A. konjac*. Quantitative real-time PCR (qRT-PCR) was employed to analyse the expression levels of the *AkCDPK* gene family under *Pectobacterium carotovorum* subsp*. carotovorum* (Pcc) stress and abiotic stressors, such as mannitol and salt. The function of *AkCDPK15* was studied by overexpression in tobacco (*Nicotiana benthamiana*). This study provides a theoretical foundation for future research on *AkCDPK* functions in *A. konjac*, potentially aiding in the development of stress-resistant konjac varieties.

## Methods

### Plant materials and treatments

*A. konjac* was supplied by the Konjac Genetic Research Center (Kunming University, Kunming, Yunnan, China). Two-month-old *A. konjac* seedlings were grown in a greenhouse at 28 ± 2 °C, 6000 lx, a relative humidity of 75%, and 16 h/8 h (day/night). Nine *A. konjac* seedlings with the same growth rate were collected from each treatment in triplicates.

For abiotic stress, two-month-old *A. konjac* seedlings were treated with 200 mM NaCl and 100 mM mannitol for 0, 24, and 48 h to induce salt and drought stress.

For biotic stress, two-month-old *A. konjac* seedlings were inoculated with either sterile water or Pcc bacterial suspension (100 µL; 10^8^ cfu/mL) [21]. The sites injected with sterile water served as controls. An inoculation site was selected for each plant. The cultivation conditions for the plants were kept unchanged. The samples were collected at 0, 24, 48, and 72 h.

### Identification of CDPKs in *A. konjac* by bioinformatic analysis

The *A. konjac* genome database was downloaded from https://doi.org/10.6084/m9.figshare.15169578. *Arabidopsis CDPK* protein sequences were downloaded from https://www.arabidopsis.org/ (S1 Table). *O. sativa CDPK* sequences were downloaded from http://rice.uga.edu/pub/data/Eukaryotic_Projects/o_sativa/annotation_dbs/pseudomolecules/version_7.0/all.dir/ (S1 Table). *Arabidopsis* and rice CDPK amino acid sequences were employed to construct a hidden Markov model to search the HMMER 3.0 (http://hmmer.janelia.org/) software [22] to identify all potential *AkCDPK* family numbers of *A. konjac*. The candidate reference sequences were compared using the BLASTP (version: ncbi last v2.10.1+) [23] with an e-value of 1e-20. The sequences containing the PF00069 and PF13499 domains were then annotated as *AkCDPK* gene family numbers by pfamscan (version: v1.6) and Pfam A (version: v33.1) [24,25] (S1 Table).

### Sequence analysis of *AkCDPK*

The bioinformatics analysis of *AkCDPK* were predicted using the ExPASY PROTPARAM tool (http://web.expasy.org/protparam/). MEME software (version: v5.0.5) [26] was used to analyse the similarity and diversity of protein motifs, and conserved motifs of AkCDPK family numbers. MG2C (http://mg2c.iask.in/mg2c_v2.1/) [27] was employed to draw the chromosome physical location map of the *AkCDPK* family numbers. The transmembrane domains, signal peptides, and subcellular localisation were predicted by DeepTMHMM (version 1.0.8), SignalP (version: v5.0b), and WOLFPSORT (https://wolfpsort.hgc.jp/), respectively. The transcription factor (TF) binding sites in each *AkCDPK* was predicted using the PlantCARE software (http://bioinformatics.psb.ugent.be/webtools/plantcare/html/) [28].

MAFFT (version: v7.427) [29] was employed to perform multiple alignments of CDPK sequences from *A. konjac* (AkCDPK), *Arabidopsis thalliana* (AtCDPK), and *Oriza sativa* (OrCDPK). MEGA (MEGA10) software was used to construct phylogenetic tree (neighbour-joining (NJ) method, 1000 bootstrap) using the p-distance model [30]. The NJ tree was then annotated by the interactive Tree of Life v6 software (https://itol.embl.de/) [31]. The AkCDPK protein multiple sequence alignment was performed using Jalview software [32] to further elucidate the characteristics of the *AkCDPK* family. And the GSDS tool (http://gsds.cbi.pku.edu.cn/) [33] was employed to display the gene structure.

### Selection pressure analysis of the *AkCDPK* family

The non-synonymous substitution rate (Ka)/ synonymous substitution rate (Ks) ratios of paralogous *AkCDPK* gene pairs were calculated using KaKs_Calculator (version: 2.0) software to determine whether *AkCDPKs* had selection pressure [34].

### Collinearity analysis of *AkCDPK*

MCScanX software was employed to conduct the collinearity analysis of *AkCDPK* [35].

### Expression profiles of *AkCDPK*

The expression profiles of *AkCDPK* in the roots, corms, petioles, and leaves were obtained from the NCBI (S2 Table). The expression levels under salt stress, drought stress and Pcc stress treatments were determined using qRT-PCR.

*A. konjac* seedlings were cultivated at 27 ± 2 °C for one months. Total RNA was extracted from the all tested samples using the Trelief ® Hi-Pure Plant RNA Plus Kit (Tsingke Biotechnology Co., Ltd., Beijing, China), the total RNA was revised to cDNA using SynScript® III cDNA Synthesis Mix (Tsingke). Followed by the 1.1 × EasyQ SYBR qPCR Mix (Low ROX Premixed) EasyQ (Tsingke) was employed to conduct qRT-PCR. All experimental protocols were based on the manufacturer’s instructions. Each reaction carried out with three technical replicates. The 2^−(ΔΔCt)^ method [36] was used to calculate relative expression levels of the tested *AkCDPK* genes. The qRT-PCR primers were shown in S3 Table.

### *AkCDPK15* subcellular localisation and phosphorylation site analyses

The sequence 35S::AkCDPK15::GLOsGFP was generated by amplifying the coding sequence of *AkCDPK15* without the terminal codon from *A. konjac* cDNA, using the primers pBWA(V)HS-AkCDPK15-GLOsGFP-F (AGAGAACACGGGGGACTTTGCAACATGGGCAACACATGCCGC) and pBWA(V)HS-AkCDPK15-GLOsGFP-R (GTACTGAAGACAGAGCTAGTTACATTAGGATGCCAATGGAGAACCTCTCATG). The PCR fragment was cloned into the pBWA(V)HS-GLOsGFP vector using the MonClone™ Single Assembly Cloning Mix (Mona [Suzhou] Biotechnology Co., Ltd., Suzhou, China). The constructed expression vector pBWA (V) HS-AkCDPK15 Glosgfp was added to GV3101 for cultivation. After expansion, the bacterial cells were collected and resuspended in a 10 mM MgCL_2_ (containing 120 μM AS) suspension (OD = 0.6–0.8). The lower epidermis of tobacco was then injected with 1 ml of the above suspension. After injection, the cells were cultured under dark conditions for two days. Labelled leaves were removed, and glass slides were observed under a laser confocal microscope and photographed.

Western blot analysis was performed on transgenic tobacco leaves using transient expression of the pBWA (V) HS-ACDPK15-Glosgfp vector (S1 Fig). The target band was cut and enzymatically hydrolysed using trypsin. The hydrolysed peptide segments were desalted and detected using high-performance liquid mass spectrometry. Finally, the mass spectrometry data were analysed using PD software with the parameter phospho for variable modification, fixed modification for carbammidomethyl (C), and trypsin/P for enzyme digestion, with the first-level mass spectrometry matching tolerance was 3 ppm; the secondary mass spectrometry matching was 20 ppm.

### *AkCDPK15* gene function analysis

To explore the function of the *AkCDPK15* gene, a plate germination experiment was conducted using transgenic lines obtained as follows.

The sequence 35S::*AkCDPK15*::GFP was generated by amplifying the coding sequence without the terminal codon of *AkCDPK15* from *A. konjac* cDNA using primers pCAMBIA-*AkCDPK15*-F (GAGGACAGGGTACCCGGGGATCCATGGGCAACACATGCCGC) and pCAMBIA-*AkCDPK15*-R (CTAGTGTCGACTCTAGAGGATCCTTAGGATGCCAATGGAGAACCTCTCATG). The PCR fragment was cloned into the *pCAMBIA-1300* vector using the MonClone™ Single Assembly Cloning Mix (Mona [Suzhou] Biotechnology Co., Ltd., Suzhou, China). Followed by transforming the successfully constructed *pCAMBIA-AkCDPK15* plasmid into *Agrobacterium tumefaciens* strain GV3101. This was followed by the transformation *of A. tumefaciens* carrying *the pCAMBIA-AkCDPK15* vector into *N. benthamiana* leaves and overexpression of *AkCDPK15* via the leaf disc transformation method. The obtained transgenic strains were amplified using specific primers, pCAMBIA-AkCDPK15-FA (TTCATTTGGAGAGAACACGGGGGAC) and pCAMBIA-AkCDPK15-RA (GATTGAGAGGAAGGACAACCAA) (S2 Fig). The overexpressing *AkCDPK15* representative lines were used for further analyses.

First, transgenic (OE1, OE2, OE3) and wild-type (WT) strains were cultured on a flat plate (130 mm*130 mm) containing ½ Murashige & Skoog (MS) medium supplemented with 200 mM NaCl and 100 mM mannitol, and a statistical analysis on the root length of *AkCDPK15* was conducted after 14 d culture (n = 10).

Next, one-month-old *AkCDPK15-*overexpressing and WT tobacco strains were subjected to water deficit treatment. Twenty tobacco plants for both *AkCDPK15* transgenic strain and WT strain were subjected to drought stress conditions. For the drought stress treatment, water was withheld from the *AkCDPK15* transgenic and WT strains for two weeks, followed by regular watering for two days. Malondialdehyde (MDA), H_2_O_2_, proline, soluble sugar accumulation, and antioxidant enzyme activity were then measured.

For H_2_O_2_ accumulation assessment, detection kits for MDA content, soluble sugar content, proline content, and superoxide dismutase (SOD), catalase (CAT), and peroxidase (POD) enzyme activities (Beijing Solarbio Technology Co., Ltd., Beijing, China) were used with spectrophotometry.

## Results

### Identification of AkCDPK family members in *A. konjac*

A total of 29 putative CDPK proteins were identified in the *A. konjac* genome and named AkCDPK1–AkCDPK29*.* The full length of the 29 AkCDPK proteins varied from 497 (AkCDPK1) to 616 (AkCDPK29) amino acids (aa), with coding sequence (CDS) lengths ranging from 1494 to 1851 bp. The MW varied from 51.80 (AkCDPK3/11) to 66.94 KDa (AkCDPK29), whereas the theoretical isoelectric points (pI) varied from 5.16 (AkCDPK2) to 9.08 (AkCDPK21). Among the family members, most genes encoded unstable protein. The grand average of hydropathy (GRAVY) varied from −0.59 to −0.186, indicating that all AkCDPK are hydrophilic. Most AkCDPK contained both the predicted N-myristoylation and S-palmitoylation sites, whereas AkCDPK3 contained only one predicted N-myristoylation site. AkCDPK1, 4, and 19 were predicted to contain no N-myristoylation or S-palmitoylation sites. All AkCDPK harboured protein kinase and EF-hand domains (Table 1).

**Table 1:** Characteristics of *AKCDPK* in *Amorphophallus konjac.*

Gene Name	Gene ID	Chromosome Location	coding sequence length	Amino acid (aa) no.	Molecular weight (Da)	Isoelectric points	GRAVY	No. of EF Hands	N-Acylation Prediction (No.)
AkCDPK1	evm.model.CTG_17200.13.1_Akon	CTG_17200:263057..267912	1494	497	56254.16	5.34	−0.339	2	0
AkCDPK2	evm.model.CTG_17475.4_Akon	CTG_17475:70008..75001	1677	558	62129.62	5.9	−0.328	2	N-Myr (1)-S-Palm (3)
AkCDPK3	evm.model.CTG_18290.6_Akon	CTG_18290:113867..118587	1380	459	51801.14	5.47	−0.345	2	N-Myr (1)–S-Palm (0)
AkCDPK4	evm.model.CTG_20042.12_Akon	CTG_20042:179353..185036	1767	588	65245.22	5.35	−0.332	2	0
AkCDPK5	evm.model.CTG_3081.5_Akon	CTG_3081:112268..117036	1626	541	60596.9	5.4	−0.435	2	N-Myr (1)–S-Palm (3)
AkCDPK6	evm.model.CTG_9134.3_Akon	CTG_9134:65417..85398	1638	545	61434.67	6.56	−0.555	2	N-Myr (1)–S-Palm (1)
AkCDPK7	evm.model.HIC_ASM_0.4363_Akon	HIC_ASM_0:209595801..209690834	1749	582	64668.29	5.95	−0.336	2	N-Myr (1)–S-Palm (2)
AkCDPK8	evm.model.HIC_ASM_0.4386_Akon	HIC_ASM_0:210725827..210794238	1665	554	62771.33	8.79	−0.59	2	N-Myr (1)–S-Palm (2)
AkCDPK9	evm.model.HIC_ASM_0.5124_Akon	HIC_ASM_0:237769900..237776387	1605	534	59417.58	5.84	−0.466	2	N-Myr (1)–S-Palm (1)
AkCDPK10	evm.model.HIC_ASM_0.5128_Akon	HIC_ASM_0:237909385..237915869	1629	542	60692.22	5.99	−0.414	2	N-Myr (1)–S-Palm (1)
AkCDPK11	evm.model.HIC_ASM_0.5318_Akon	HIC_ASM_0:243665230..243669940	1494	497	56254.16	5.34	−0.339	2	N-Myr (1)–S-Palm (0)
AkCDPK12	evm.model.HIC_ASM_0.5386_Akon	HIC_ASM_0:245323336..245339406	1629	542	60526.93	5.16	−0.296	2	N-Myr (1)–S-Palm (1)
AkCDPK13	evm.model.HIC_ASM_1.10394.2_Akon	HIC_ASM_1:473829711..473843511	1629	542	60450.88	5.19	−0.292	2	N-Myr (1)–S-Palm (1)
AkCDPK14	evm.model.HIC_ASM_1.1830_Akon	HIC_ASM_1:50282319..50299659	1647	548	62047.69	9.08	−0.514	2	N-Myr (1)–S-Palm (1)
AkCDPK15	evm.model.HIC_ASM_11.3224_Akon	HIC_ASM_11:79853509..79858908	1602	533	60074.41	6.02	−0.505	2	N-Myr (1)–S-Palm (1)
AkCDPK16	evm.model.HIC_ASM_11.5617_evm.model.HIC_ASM_11.5618_Akon	HIC_ASM_11:206243534..206313183	1602	533	60060.38	6.02	−0.506	2	N-Myr (1)–S-Palm (1)
AkCDPK17	evm.model.HIC_ASM_4.1275_Akon	HIC_ASM_4:33212080..33215666	1683	560	63368.4	6.25	−0.483	2	N-Myr (1)–S-Palm (1)
AkCDPK18	evm.model.HIC_ASM_4.2160.1_Akon	HIC_ASM_4:61367073..61413550	1647	548	62028.64	9.01	−0.512	2	N-Myr (1)–S-Palm (2)
AkCDPK19	evm.model.HIC_ASM_4.2288.1_Akon	HIC_ASM_4:64437246..64442101	1713	570	64886.09	6.81	−0.494	2	0
AkCDPK20	evm.model.HIC_ASM_4.2664_Akon	HIC_ASM_4:74975034..74980004	1602	533	60057.74	5.91	−0.434	2	N-Myr (1)–S-Palm (3)
AkCDPK21	evm.model.HIC_ASM_5.10001_Akon	HIC_ASM_5:430159214..430172624	1713	570	64818.01	6.81	−0.487	2	N-Myr (1)–S-Palm (2)
AkCDPK22	evm.model.HIC_ASM_5.11603_Akon	HIC_ASM_5:468342621..468347497	1851	616	66940.77	4.85	−0.242	2	N-Myr (1)–S-Palm (2)
AkCDPK23	evm.model.HIC_ASM_5.11616_Akon	HIC_ASM_5:468702494..468707370	1380	459	51801.14	5.47	−0.345	2	N-Myr (1)–S-Palm (2)
AkCDPK24	evm.model.HIC_ASM_5.8866_Akon	HIC_ASM_5:402294432..402317205	1563	520	57603.92	5.86	−0.186	2	N-Myr (1)–S-Palm (4)
AkCDPK25	evm.model.HIC_ASM_5.9948_Akon	HIC_ASM_5:429025695..429046511	1599	532	59535.98	6.15	−0.453	2	N-Myr (1)–S-Palm (2)
AkCDPK26	evm.model.HIC_ASM_9.420_Akon	HIC_ASM_9:11640424..11657737	1566	521	58269.39	6.13	−0.448	2	N-Myr (2)–S-Palm (3)
AkCDPK27	evm.model.HIC_ASM_9.462_Akon	HIC_ASM_9:12209941..12217085	1638	545	60892.25	6.12	−0.457	2	N-Myr (1)–S-Palm (2)
AkCDPK28	evm.model.HIC_ASM_9.721_Akon	HIC_ASM_9:17049898..17063692	1632	543	60748.12	6.12	−0.456	2	N-Myr (2)–S-Palm (3)
AkCDPK29	evm.model.HIC_ASM_9.7625_Akon	HIC_ASM_9:356624016..356745767	1677	558	62129.62	5.9	−0.328	2	N-Myr (1)–S-Palm (1)

### Phylogenetic, gene structure, and chromosomal distribution of *AkCDPK*

**Fig 1:**
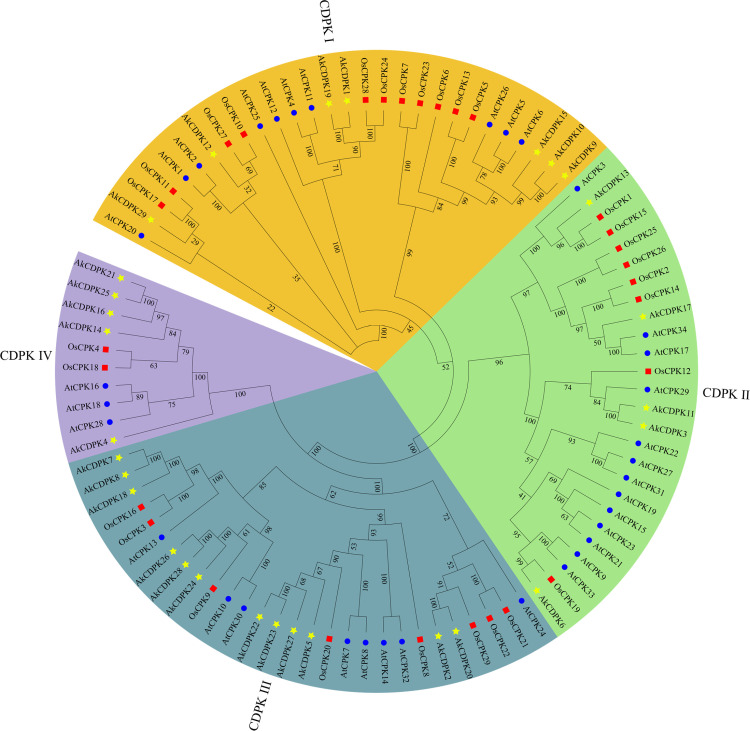
Phylogenetic relationships of CDPK proteins in *Amorphophallus konjac* (yellow pentagram), *Arabidopsis thaliana* (blue circle), and *Oryza sativa* (red box). The evolutionary tree was constructed using MEGA10 software with the neighbour-joining method. The numbers next to the branch show 1000 bootstrap replicates, expressed as percentages. The phylogenetic groups of AkCDPK are marked with different colours and legends.

To understand the phylogenetic relationships among the CDPKs in *A. konjac*, CDPK sequences of three species, *A. konjac*, *A. thaliana*, and *O. sativa*, were employed to construct NJ tree using MEGA10. The CDPK sequences grouped into four subfamilies, with 7, 5, 12, and 5 members in Groups I, II, III, and IV, respectively (Fig 1).

### Gene structure, motif, and chromosomal location analyses of *CDPK* in *A. konjac*

**Fig 2:**
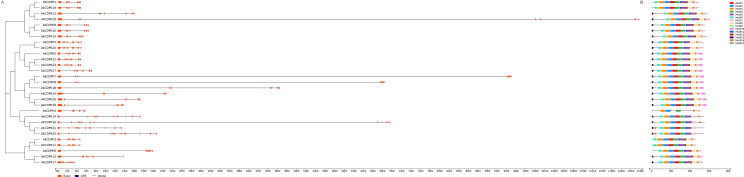
Phylogenetic relationships, gene structure, and conserved motifs of Ak*CDPK.* **(A)** Structure of the *AkCDPK* genes. Orange, black, and blue boxes represent the exons, introns, and untranslated regions, respectively. **(B)** Conserved motifs of AkCDPK proteins. The motifs are indicated by different coloured boxes and their numbers are listed on the right.

To understand the possible structural evolution of *AkCDPK*, the diversity of the exon-intron organisation within the *AkCDPK* family was compared. As shown in Fig 2A, all *AkCDPK* members possessed 5–11 introns (3 with 5 introns, 9 with 6 introns, 13 with 7 introns, and 4 with 11 introns). In Group I, members had seven or eight exons. A diverse number of exons were found in Group II: six exons were found in *AkCDPK24*, *AkCDPK26*, and *AkCDPK28*; seven exons were found in *AkCDPK7*, *AkCDPK8*, and *AkCDPK18*; and eight exons were found in *AkCDPK2*, *AkCDPK20*, *AkCDPK5*, *AkCDPK22*, *AkCDPK23*, and *AkCDPK27.* The members in Group III had 7 or 12 exons, of which 7 exons were found in *AkCDPK4* and 12 were found in *AkCDPK14*, *AkCDPK16*, *AkCDPK21*, and *AkCDPK25*. In Group IV, all members had eight exons. These results indicated that *CDPK* with higher homogeneity usually had the same number of exons.

The MEME software was used to further understand the similarity and diversity of AkCDPK protein motifs. Fifteen conserved motifs were predicted in AkCDPK family numbers with similar motif types and sequences. Hence, the 29 AkCDPK proteins had the same conserved motifs and orders. AkCDPK4 contained six motifs. AkCDPK1, AkCDPK19, AkCDPK4, AkCDPK3, AkCDPK11, and AkCDPK16 did not contain a motif 13. Compared to other family members, AkCDPK5, AkCDPK22, AkCDPK23, AkCDPK27, AkCDPK7, AkCDPK8, AkCDPK18, AkCDPK24, AkCDPK26, and AkCDPK28 contained a unique motif 10 (Fig 2B).

**Fig 3:**
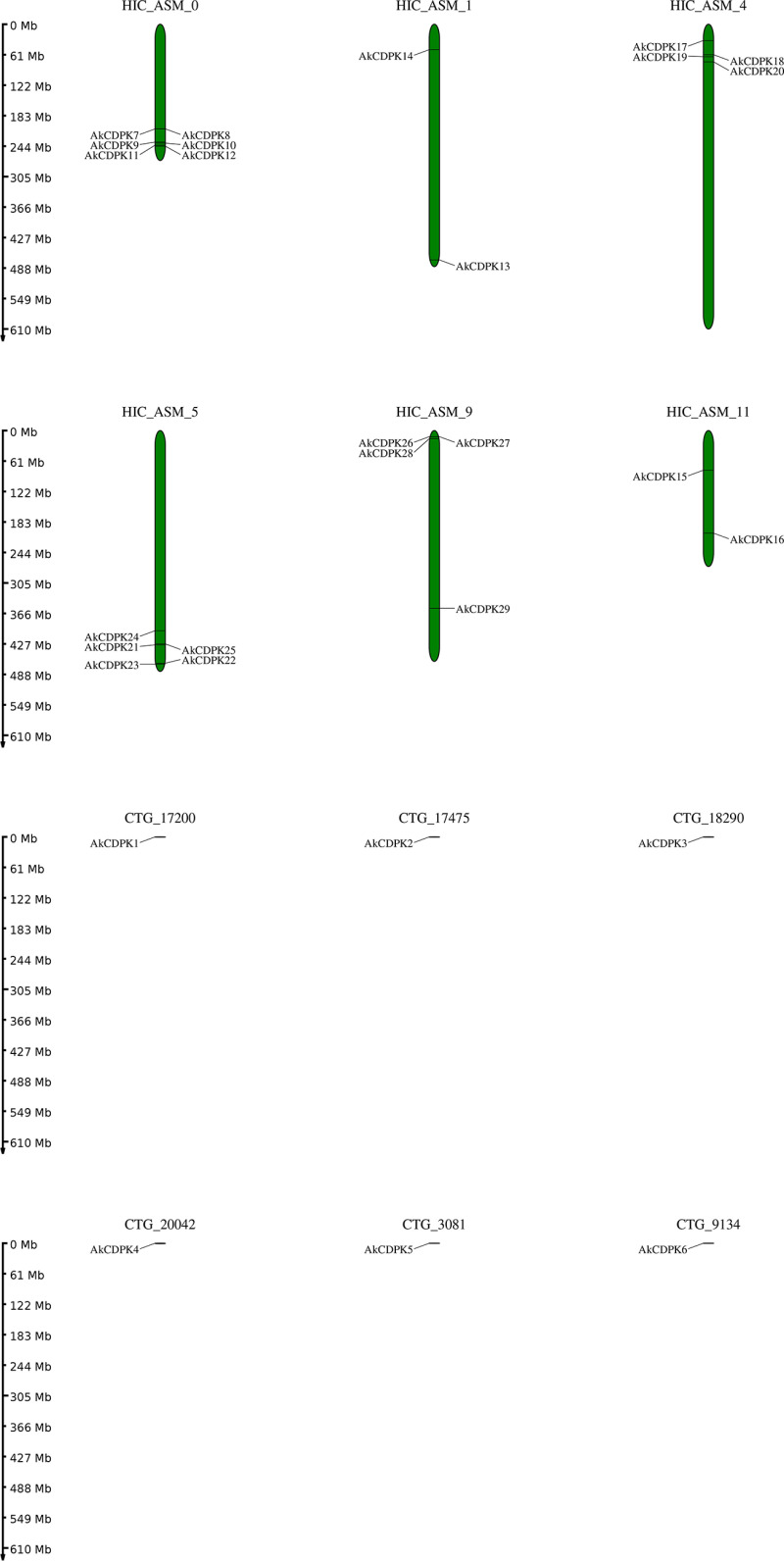
Physical map of *AkCDPK* chromosome locations. The vertical axis represents the length of the chromosomes.

All 29 *AkCDPK* in *A. konjac* were mapped to 12 chromosomes (Fig 3). And there were significant differences in the number of genes on different chromosomes. The largest numbers of genes (*AkCDPK7, AkCDPK8, AkCDPK9, AkCDPK10, AkCDPK11, AkCDPK12*) were located on chromosome HIC-ASM-0; five genes (*AkCDPK21, AkCDPK22, AkCDPK23, AkCDPK24, AkCDPK25*) were located on chromosome HIC-ASM-5, and chromosomes HIC-ASM-4 and HIC-ASM-9 were found to contain four *AkCDPK* each. Chromosomes HIC-ASM-1 and HIC-ASM-11 contained two *AkCDPK*. Chromosomes CTG-17200, CTG-17475, CTG-18290, CTG-20042, CTG-3081, and CTG-9134 harboured only one *AtCDPK* each.

**Fig 4:**
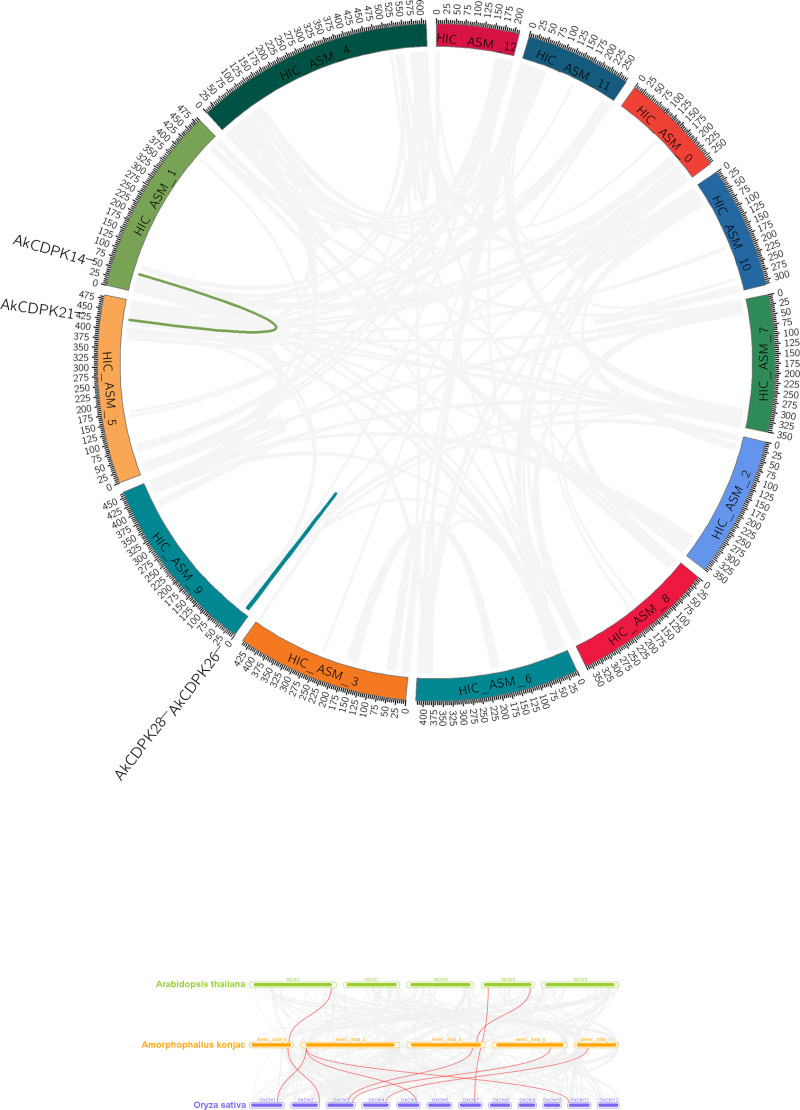
Synteny analysis of *CDPK* in *Amorphophallus konjac*, *Arabidopsis thaliana*, and *Oryza sativa.* **(A)** Schematic representation of the chromosomal distribution and intrachromosomal relationships of *A. konjac CDPK* genes. **(B)** Collinear correlation between *A. konjac* and *A. thaliana,* and *O. sativa*. Grey lines in the background indicate the collinear blocks within *A. konjac* and other plant genomes, whereas the red lines highlight syntenic *AkCDPK* gene pairs.

To elucidate the replication events of the *AkCDPK* gene family, the MCScanX software was used for collinearity analysis. Two duplicated *CDPK* gene pairs were found in the *A. konjac* genome: one was segmentally duplicated (*AkCDPK14/21*) and the other was tandemly duplicated (*AkCDPK26/28*) (Fig 4A). Furthermore, the collinear correlation among *A. konjac, A. thaliana*, and *O. sativa* was calculated to infer the evolutionary history of *CDPKs.* Two *AkCDPK* showed syntenic relationships with those in *Arabidopsis* and six with those in rice (Fig 4B; S4 Table). Two pairs of syntenic orthologous genes (one-to-one) were identified between *Arabidopsis* and *A. konjac CDPK* genes: *AkCDPK11*-*AtCPK29* and *AkCDPK21*- *AtCPK18* (Fig 4B; S4 Table). Between rice and *A. konjac CDPK*, there were two kinds of syntenic orthologous gene pairs: One *A. konjac* gene and multiple rice genes, such as *AkCDPK17*-*OsCPK2/14/25*, and one *A. konjac* gene vs. one rice gene, such as *AkCDPK9*-*OsCPK6*, *AkCDPK15*-*OsCPK13*, *AkCDPK24*-*OsCPK9*, and *AkCDPK29*- *OsCPK11* (Fig 4B; S4 Table), indicating that these genes might have been derived from the same ancestor of rice and *A. konjac.* For further evolutionary studies, the number of Ka and Ks were computed to analyse selection pressure (S5 Table). Most orthologous *CDPK* gene pairs had Ka/Ks < 1, indicating those *AkCDPK* numbers underwent strong purifying selective pressure during evolution.

**Fig 5:**
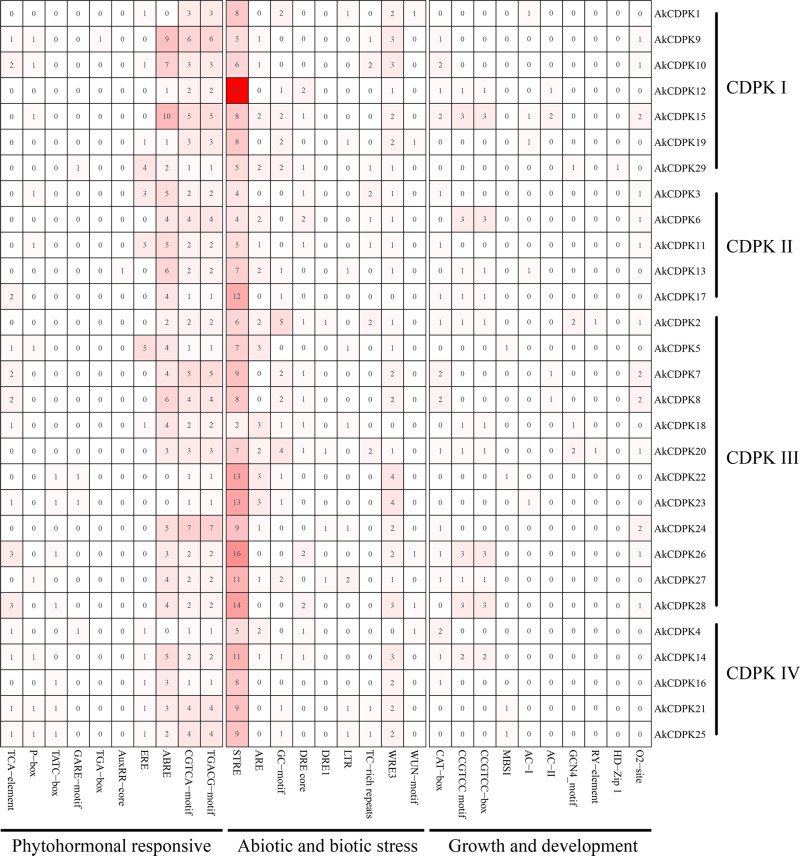
Cis-acting regulatory elements in the *AkCDPK* promoter region.

To explore which factors might regulate the level of *AkCDPK* expression, the PlantCARE database was used to predict cis-acting regulatory elements of *AkCDPK* (S6 Table). CAAT-box, activating sequence 1 (as-1), and TATA box are cis-acting elements shared by all *AkCDPK* genes. The remaining cis-acting elements of the genes can be divided into four categories. First, there are multiple phytohormone responsive elements, including salicylic acid response element (TCA-element), gibberellin-responsive element (P-box, TATC-box, and GARE-motif), auxin-responsive element (TGA-box, AuxRR-core), ethylene-responsive element (ERE), ABA response element (ABRE), and MeJA-responsive element (CGTCA-motif and TGACG-motif), suggesting that *AkCDPK* expression might be regulated by multiple phytohormones. Multiple elements were involved in the light response, such as the G-box, Box 4, and TCCC-motif. What’s more there are varieties of growth and development elements, including meristem expression elements (CAT-box, CCGTCC motif, and CCGTCC-box), flavonoid biosynthetic gene regulation elements (MBSI), lignin biosynthetic gene regulation elements (AC-I and AC-II), endosperm expression elements (GCN4_motif), seed-specific regulatory elements (RY-element), palisade mesophyll cell elements (HD-Zip 1), and circadian elements (O2-site). In addition, stress-responsive elements were found in *AkCDPK*, for example, the anaerobic induction element (ARE and GC-motif), dehydration-responsive element (DRE core and DRE1), low-temperature-responsive elements, defence and stress-responsive elements (TC-rich repeats), stress-responsive elements (STRE), and wound-responsive elements (WRE3 and WUN-motif) (Fig 5, S6 Table). There were also some transcription factor-binding elements, including the MYB-like sequence, Myb-binding site, and W-box (S6 Table). ABRE, MeJA responsiveness, ARE, and STRE elements were detected in the promoter regions of most *AkCDPK*. The above results suggest that *AkCDPK* are not only involved in the growth and development of *A. konjac* but also in its response to various environmental stresses.

### Expression profiles of *AkCDPK* in different tissues

**Fig 6:**
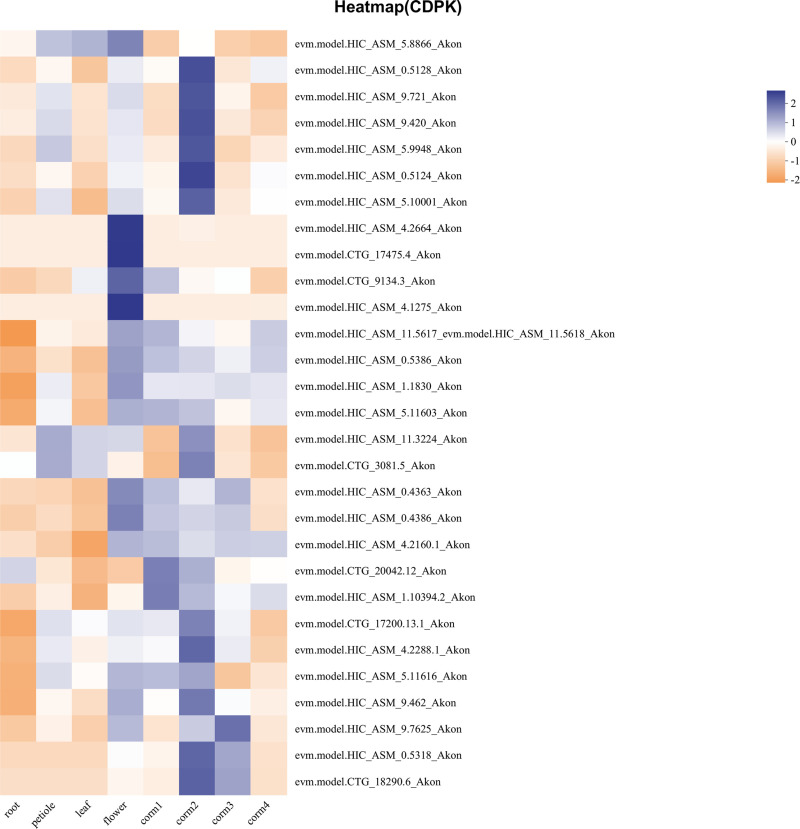
Expression patterns of *AkCDPK* in different tissues (root, bulb, petiole and leaf). The heat map shows the relative expression after log10 transformation.

To understand the possible roles of *AkCDPK*, the spatial expression patterns of the 29 *AkCDPK* genes were analysed using RNA-Seq expression data from *A. konjac* recently published by Li et al. [19]. The results showed that *AkCDPK2*, *3*, *11*, *17*, and *20* were not expressed in the root, bulb, leaf, or petiole. The remaining genes had high expression levels in the petiole. Moreover, *AkCDPK8, 9*, 12, and *29* transcript levels in bulbs were significantly higher than those of other genes. Compared with other genes, *AkCDPK6,* and *AkCDPK24* showed higher transcription levels in leaves (Fig 6). These results indicate that *AkCDPK* gene family members play different regulatory roles in the growth and development of *A. konjac.*

### Expression profiles of *AkCDPK* in response to biotic and abiotic stress

**Fig 7:**
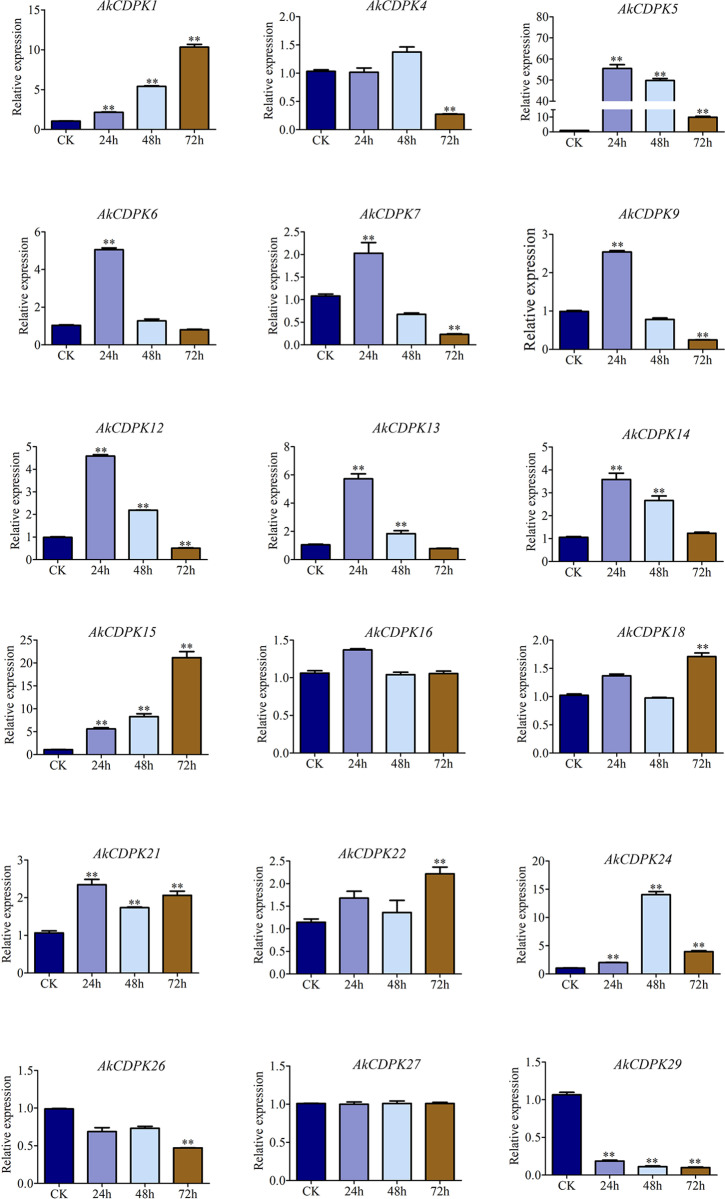
Expression profiles of *AkCDPK* during *Pectobacterium carotovorum* subsp*. carotovorum* (Pcc) infection.

**Fig 8:**
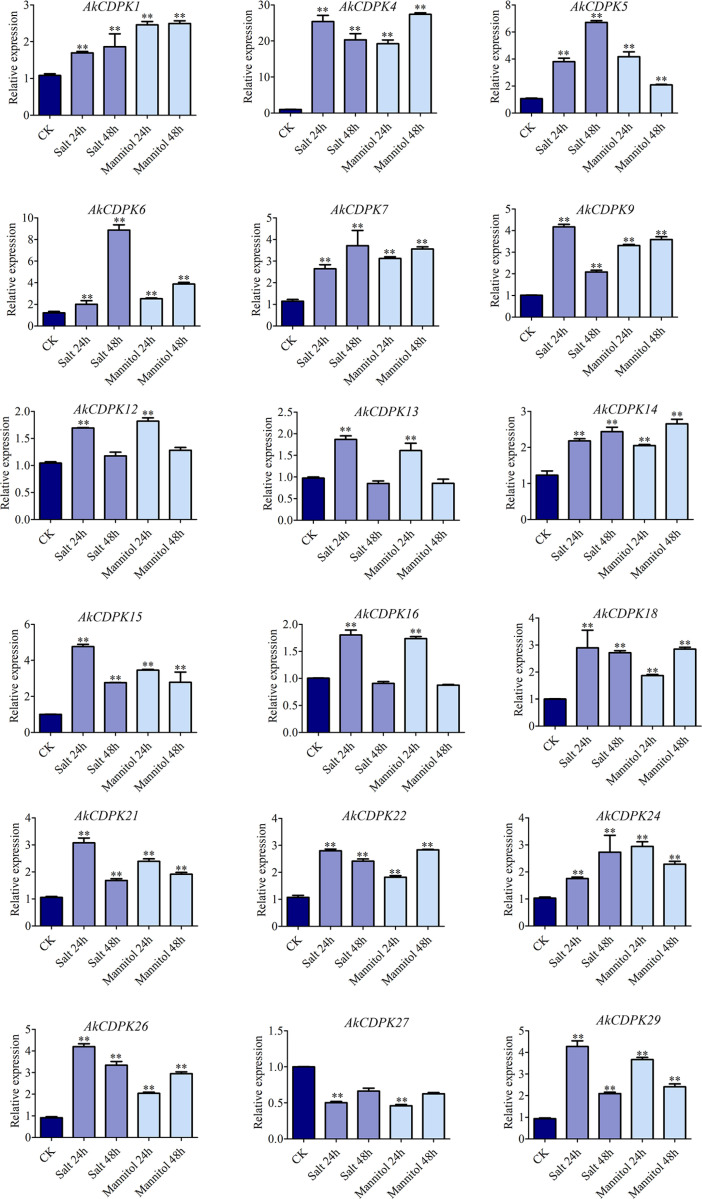
Expression profiles of *AkCDPK* under salt and mannitol stress.

To explore the expression characteristics of *AkCDPK* family members in response to external stress, qRT-PCR was used to analyse the expression patterns of 18 family members under Pcc infection, drought, and salt stress. Most *AkCDPK* family members were induced under Pcc stress, except for *AkCDPK16* and *AkCDPK27*; *AkCDPK22* and *AkCDPK18* were induced at the late stage of Pcc infection (72 h). Most of the genes were induced at the early stage of Pcc infection (24 h) and then downregulated, such as *AkCDPK1/5/6/7/9/12/13/14*. The expression levels of *AkCDPK15*, *AkCDPK21,* and *AkCDPK24* were significantly increased within 72 h of Pcc infection (Fig 7). We found that all 18 *AkCDPK* analysed responded to mannitol and salt stress. Among these genes, the expression level of *AkCDPK27* was suppressed with mannitol and salt treatment for 24 h. When *A. konjac* was subjected to mannitol and salt stress for 24 h, the expression levels of *AkCDPK12/13/16* were significantly upregulated, indicating that these three genes may participate in the early stages of osmotic stress. The expression levels of *AkCDPK1/4/5/6/7/9/14/15/18/21/22/24/26/29* were significantly higher than those in the control group within 48 h of mannitol and salt treatment (Fig 8).

### Cloning and characterisation of *AkCDPK15* in *A. konjac*

**Fig 9:**
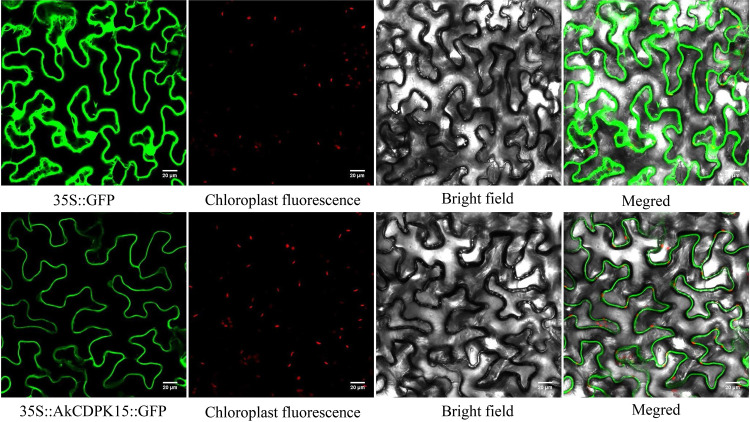
Subcellular localisation of AkCDPK15.

The expression profile of *A. konjac CDPK* family genes suggested that they play an important role in response to different biotic and abiotic stresses. To further understand their function, a Group I member (*AkCDPK15*) was selected for further study. An open reading frame (ORF) of *AkCDPK15*, which was 1749 bp long and encoded 582 amino acids, was obtained from *A. konjac*. The sequence alignment of *AtCDPK5/26* revealed a high homology with *AkCDPK15*. To characterise the subcellular localisation of AkCDPK15, pBWA(V)HS-AkCDPK15-GLOsGFP fusion protein was transformed into tobacco protoplasts. The result showed that AkCDPK15 was mainly localised in the cell membrane region, with a small amount expressed in the nucleus (Fig 9), indicating that AkCDPK15 mainly functioned at these two sites.

Because the function of *CDPK* genes is often accompanied by protein phosphorylation, we identified the phosphorylation sites of AkCDPK15. The results showed that AkCDPK15 contained eight phosphorylation sites (S87 [88.5], S92 [88.5], T94 [88.5], S359 [77.3], S436 [96.2], S453 [99.7], S508 [83.9], and T526 [90]), and the probability of all phosphorylisable amino acid sites occurring was above 75% (S3 Fig, S7 Table).

### *AkCDPK15* overexpression enhanced drought and salt tolerance

**Fig 10:**
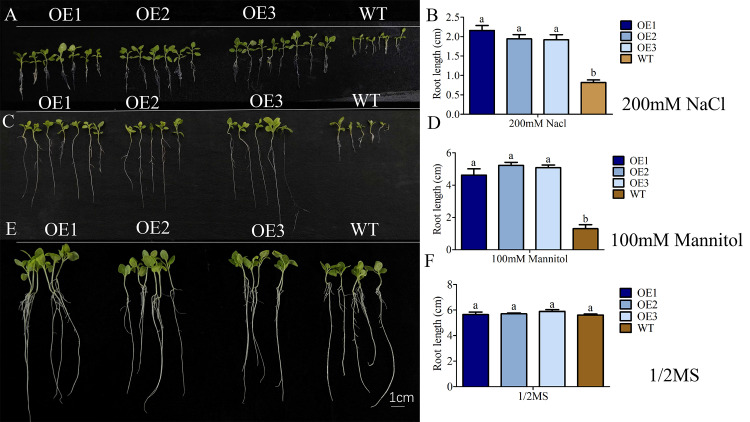
Overexpression of *AkCDPK15* improves tolerance of tobacco to drought and salt stress. **(A)** *AkCDPK15-*overexpressing and wild-type strains grown for 14 days on standard 1/2 Murashige and Skoog (MS) medium supplemented with 200 mM NaCl. **(B)** The root length bar chart of *AkCDPK15-*overexpressing and wild-type strains grown for 14 days on standard 1/2 MS medium supplemented with 200 mM NaCl. **(C)** *AkCDPK15-*overexpressing and wild-type strains grown for 14 days on standard 1/2 MS medium supplemented with 100 mM mannitol. **(D)** The root length bar chart of *AkCDPK15-*overexpressing and wild-type strains grown for 14 days on standard 1/2 MS medium supplemented with 100 mM mannitol. **(E)** *AkCDPK15-*overexpressing and wild-type strains grown for 14 days on standard 1/2 MS medium without supplementation. **(F)** The root length bar chart of *AkCDPK15* gene overexpression strains and wild-type strains grown for 14 days on standard 1/2 MS medium without supplementation. Different lowercase letters indicate significant differences, as calculated by Student’s *t*-test).

To understand *AkCDPK15* function under drought and salt conditions, we generated several overexpressing transgenic tobacco lines. Plants transformed with the empty vector were considered WT. Three *AkCDPK15-*overexpressing strains (OE1, OE2, and OE3) were used in further experiments. The root elongation experiments showed that the roots of the WT strain were significantly shorter than those of the *AkCDPK15*-overexpressing strains treated with 100 mM mannitol and 200 mM NaCl (Fig 10A, B, C, D). In contrast, there was no difference in the root length between the WT and untreated *AkCDPK15*-overexpressing strains (Fig 10E, F).

**Fig 11:**
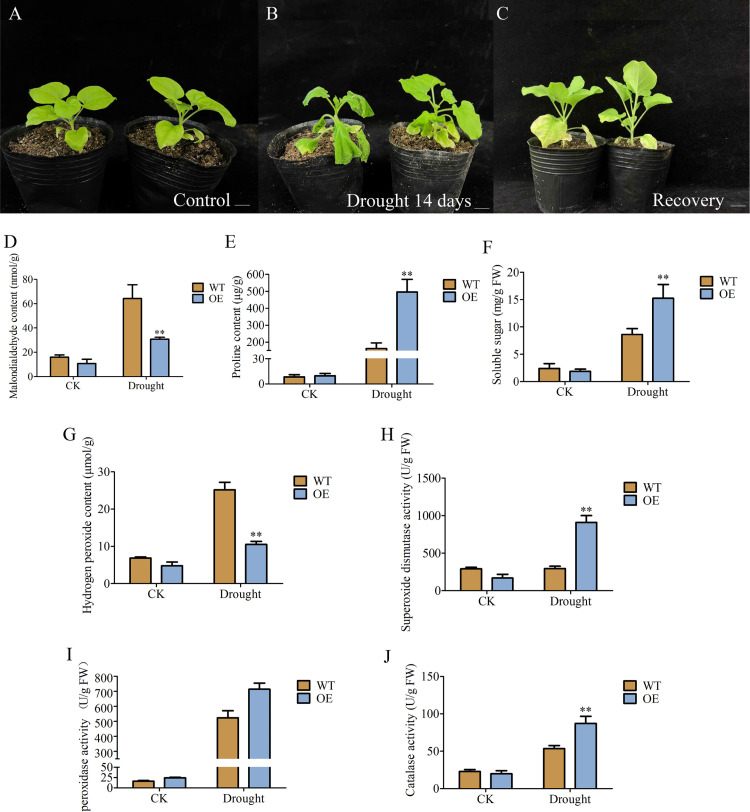
Phenotypes and physiological indices of *AkCDPK15*-overexpressing tobacco strains under normal and drought stress conditions. **(A)** One-month-old WT strains grown under normal conditions. **(B)** *AkCDPK15-*overexpressing and WT strains after 14 days without water. **(C)** *AkCDPK15-*overexpressing and WT strains in (B) after re-watering for two days. **(D-J)** The content of malondialdehyde (MDA) **(D)**, proline **(E)**, soluble sugar **(F)**, H_2_O_2_ (G) in WT and *AkCDPK15-*overexpressing strains under normal and drought stress. The activity of superoxide dismutase (SOD) **(H)**, peroxidase (POD) **(I)**, and catalase (CAT) (J) in WT and transgenic lines under normal and drought stress conditions. Data are presented as mean ± SD (n = 3, *P < 0.05; **P < 0.01, Student’s *t*-tes*t*).

To further explore the function of *AkCDPK15*, one-month-old *AkCDPK15* transgenic and WT strains were subjected to water deficit stress. Under normal growth conditions, there were no significant differences in the growth of *AkCDPK15* transgenic and WT strains (Fig 11A). After 14 days of drought treatment, the degree of leaf wilting in the WT strain was more severe than that in *AkCDPK15-*overexpressing strains (Fig 11B). Two days after re-watering, both the WT and *AkCDPK15*-overexpressing strains showed growth recovery. However, yellowing and wilting of the bottom leaves of the WT strains were still higher than those of the *AkCDPK15*-overexpressing strains (Fig 11C). Taken together, *AkCDPK15-*overexpressing strains showed stronger resistance to drought stress than did the WT strains.

To understand the physiological changes in *AkCDPK15*-overexpressing and WT strains under drought stress, the MDA, proline, soluble sugar, and H_2_O_2_ contents, as well as the SOD, POD, and CAT activities of the two strains after 14 days of water deficit were detected. The results showed that after 14 days of drought stress, the MDA content of the WT strain was approximately 2.08 times higher than that of the *AkCDPK15*-overexpressing strain (Fig 11D). The proline and soluble sugar contents of the *AkCDPK15-*overexpressing strain were approximately 3.03 and 1.77 times those of the WT strain, respectively (Fig 11E, F). The H_2_O_2_ content of the WT strain was approximately 2.39 times that of the *AkCDPK15-*overexpressing strain (Fig 11G). However, the SOD, POD, and CAT activities of the *AkCDPK15-*overexpressing strain were 3.09, 2, and 1.63 times higher than those of the WT strain, respectively (Fig 11H, I, G). No significant differences in physiological indicators were detected under normal cultivation conditions (Fig 11). In short, overexpression of *AkCDPK15* in tobacco can enhance tobacco resistance to drought stress by reducing membrane damage in plant cells, enhancing antioxidant enzyme activity, and increasing intracellular stress resistance.

## Discussion

Konjac corms are rich in glucosinolates and have broad application prospects in food, health, biomedicine, and industry, making them highly valuable crops. However, konjac is often affected by a variety of biotic and abiotic stressors, leading to a significant decline in its yield and quality. CDPK family genes not only exist in various plant tissues, such as roots, stems, and flowers, but also participate in various plant growth and development processes and respond to environmental stresses. In this study, a screening for *CDPK* family members from the *A. konjac* genome resulted in the identification of 29 members, divided into four subfamilies (Groups I-IV), together with an analysis of their structure, evolution, and expression profiles in response to phytohormones, and drought, salt, and Pcc stress.

Previous studies on evolutionary analysis of *CDPK* family members in *Arabidopsis* [37], rice [38,39], and patchouli [40] found that they are often divided into four subgroups. Similarly, the 29 *AkCDPK* family genes were divided into four subgroups. This indicates some degree of similarity in the evolution of the *CDPK* family members and in the diversity of gene structures among different species. In addition, we also found that *AkCDPK* family members contain 5–11 introns, similar to *Medicago truncatula* [41] and pineapple [42]. The number of introns among members of *AkCDPK* Group III in particular showed significant variability. Nuruzzaman et al. published that the rate of intron loss is higher than intron acquisition [43]. Therefore, we hypothesised that *AkCDPK* Group III may contain the original genes. Moreover, we noticed that in Group III, *AkCDPK* members contained a unique conserved motif 10, indicating the conservation of gene structural evolution in Group III.

The distribution of *CDPK* family members on plant chromosomes is not uniform. For example, in tomato they are mainly distributed on chromosomes 1, 10, and 11 [44], whereas in cucumber they are mainly distributed on chromosomes 2 and 5 [45]. In this study, *AkCDPK* family members were mainly distributed on chromosomes HIC_ASM_0, HIC_ASM_4, and HIC_ASM_5, but not on every chromosome. Gene replication events lead to fragment and tandem duplications, which play crucial roles in genome rearrangement and expansion [46]. In this study, there were only two collinear gene pairs within the family, indicating that *AkCDPK* had fewer copies or that copies were discarded more frequently during evolution. To further study the homologous relationships of *AkCDPK* genes in plants, an interspecific collinearity analysis was performed on *Arabidopsis* and rice. Two *A. konjac* genes (*AkCDPK11* and *AkCDPK21*) and two *Arabidopsis* genes were identified as syntenic orthologs between *A. konjac* and *Arabidopsis*, five *A. konjac* genes (*AkCDPK9*, *AkCDPK15*, *AkCDPK17*, *AkCDPK24*, and *AkCDPK29*), and seven rice genes were identified as syntenic orthologs between *A. konjac* and rice and these genes were distributed in four subgroups. We speculate these seven genes play crucial roles in the evolution of the *AkCDPK* family. Genetic evolutionary selection analysis revealed that most *AkCDPK* were purified and selected, which was consistent with the results obtained for Chinese cabbage (*Brassica rapa*) [47] and *M. truncatula* [41].

There were multiple cis-acting elements in *AkCDPK* family member promoter regions that responded to plant hormones, stress, growth, and development, suggesting that the expression of *AkCDPK* may be regulated by phytohormones and stress, as well as being related to plant growth and development. This finding is consistent with the studies on *Arachis hypogaea* [48] and *Hevea brasiliensis* [42]. Moreover, RNA-Seq analysis of *AkCDPK* in different tissues revealed tissue specificity in *AkCDPK* expression. For example, *AkCDPK2*, *AkCDPK20*, and *AkCDPK17* were not expressed in the leaf, petiole, root and corm of *A. konjac*, and the *AkCDPK17* homologous genes *AtCPK17* and *AtCPK34* are involved in the development of *Arabidopsis* pollen tubes [49], whereas the *AoCPK16* gene (*AoCPK16* gene is homologous to *AtCPK17* and *AtCPK34*) has high expression levels in pineapple flowers and leaves [42]. *CDPK* is also involved in the response to various environmental stressors. For example, in *Arabidopsis*, under cold stress, the AtCPK28 phosphorylation cascade is activated to regulate transcriptional reprogramming downstream of Ca^2+^ signalling, positively regulating the response to cold stress [50]. In wheat, the *TaCDPK27* gene enhanced resistance to salt stress by reducing ROS production, increasing antioxidant enzyme (POD, SOD, and CAT) activity, and reducing damage to photosystem II [51]. Similarly, in potatoes, *StCDPK2* actively participates in salt resistance by enhancing the antioxidant system [52]. In rape (*Brassica napus*), BnaCPK5 interacts with the ABA-responsive element-binding factors, BnaABF3 and BnaABF4, enhancing its drought resistance [53]. In rice, *OsCPK10* positively mediates plant tolerance to drought and rice blast disease by enhancing their antioxidant capacity and protecting them from ROS damage [54]. In this study, all 18 tested genes were induced by mannitol and salt, and 16 genes were induced by Pcc. The expression level of the Group I gene *AkCDPK15* was significantly altered under mannitol-, salt-, and Pcc-induced stress. Therefore, the ORF sequence of *AkCDPK15* was cloned and its response to drought and salt was analysed using tobacco *AkCDPK15-*overexpressing tobacco strains. Plants under drought stress often experience changes in a series of physiological indicators, such as cell membrane damage, accumulation of oxides, and changes in enzyme activity [55,56]. A dot plate experiment showed that the root length of *AkCDPK15-*overexpressing strains was significantly longer than that of the WT strain under mannitol (100 mM) and salt (200 mM NaCl) stress for 14 days. Furthermore, after 14 days water deficit, the activity of POD, SOD, and CAT and the content of proline and soluble sugar of the *AkCDPK15*-overexpressing strains were significantly higher than those of the WT strain, whereas the MDA and H_2_O_2_ contents were significantly decreased compared to those of the WT strains. Under normal cultivation conditions, there were no significant differences in these physiological indicators between the *AkCDPK15*-overexpressing and WT strains. Taken together, these findings show that *AkCDPK15* positively regulates drought and salt resistance in transgenic tobacco.

## Conclusion

In brief, 29 *AkCDPK* genes of *A. konjac* were identified, which could be divided into four subgroups and were unevenly distributed on 12 chromosomes. The gene motifs of these *AkCDPK* genes were highly conserved. There was one segmentally duplicated gene pair and one tandemly duplicated gene pair in the *AkCDPK* family. Between *A. konjac* and *Arabidopsis*, there were two syntenic blocks from CDPKs and six between *A. konjac* and rice. Furthermore, most *AkCDPK* genes underwent purifying selection during the evolutionary process. What’s more, based on the predicted *AkCDPK* promoters, those *AkCDPK* genes are related to phytohormone induction, defence and stress responses, and other functions. *AkCDPK* exhibited tissue specificity, and the qRT-PCR analysis showing that *AkCDPK* responded to salt, drought, and Pcc stress. Finally, *AkCDPK15* was cloned, and shown to positively regulate plant drought and salt tolerance.

## Supporting information

S1 Table CDPK amino acid sequences of *A. thaliana*, *O. sativa*, and *A. konjac.* (XLSX)

S2 Table NCBI accession IDs for the transcriptome of *A. konjac.* (XLSX)

S3 Table qRT-PCR primers. (XLSX)

S4 Table Duplications of *CDPK* genes among *A. konjac*, *A. thaliana,* and *O. sativa.* (XLSX)

S5 Table Ka/Ks of the *AkCDPK* family. (XLS)

S6 Table Annotation of cis-acting regulatory elements in *AkCDPK* promoters. (XLSX)

S7 Table AkCDPK15 phosphorylation site information. (XLSX)

S1 Fig Western blot gel electrophoresis of *AkCDPK15.* (TIF)

S2 Fig Gel electrophoresis of identification of *AkCDPK15* transgenic strains. (TIF)

S3 Fig Identification map of phosphorylation sites in AkCDPK15. (TIF)

## Abbreviations

ABA: abscisic acid
ATP: adenosine triphosphate
BLASTP: basic local alignment search tool for proteins
CaMs: calmodulins
CAT: catalase
CDPKs: calcium-dependent protein kinases
CBD: calcium-binding domain
FPKM: fragments per kilobase of transcript per million mapped reads
GFP: green fluorescent protein
GRAVY: grand average of hydropathy
GTP: guanosine triphosphate
GSDS: gene structure display server
iTOL: Interactive Tree Of Life
JA: jasmonic acid
MS: Murashige and Skoog
MW: molecular weight
OE: overexpression
ORF: open reading frame
Pcc: *Pectobacterium carotovorum* subsp. *Carotovorum*
Pfam: protein families database
PI: isolectric point
PKD: protein kinase domain
POD: peroxidase
qRT-PCR: quantitative real-time PCR
ROS: reactive oxygen species
SA: salicylic acid
SOD: superoxide dismutase
VNTD: N-terminal variable domain
WT: wild type

## References

[1] Peng Y, van Wersch R, Zhang Y. Convergent and Divergent Signaling in PAMP-Triggered Immunity and Effector-Triggered Immunity. Mol Plant Microbe Interact. 2018;31(4):403–9. doi: 10.1094/MPMI-06-17-0145-CR 29135338

[2] Thulasi Devendrakumar K, Li X, Zhang Y. MAP kinase signalling: interplays between plant PAMP- and effector-triggered immunity. Cell Mol Life Sci. 2018;75(16):2981–9. doi: 10.1007/s00018-018-2839-3 29789867 PMC11105241

[3] Kopecká R, Kameniarová M, Černý M, Brzobohatý B, Novák J. Abiotic Stress in Crop Production. Int J Mol Sci. 2023;24(7):6603. doi: 10.3390/ijms24076603 37047573 PMC10095105

[4] Agurla S, Gahir S, Munemasa S, Murata Y, Raghavendra AS. Mechanism of Stomatal Closure in Plants Exposed to Drought and Cold Stress. Adv Exp Med Biol. 2018;1081:215–32. doi: 10.1007/978-981-13-1244-1_12 30288712

[5] Harper JF, Breton G, Harmon A. Decoding Ca(2+) signals through plant protein kinases. Annu Rev Plant Biol. 2004;55:263–88. doi: 10.1146/annurev.arplant.55.031903.141627 15377221

[6] Ludwig AA, Romeis T, Jones JDG. CDPK-mediated signalling pathways: specificity and cross-talk. J Exp Bot. 2004;55(395):181–8. doi: 10.1093/jxb/erh008 14623901

[7] Atif RM, Shahid L, Waqas M, Ali B, Rashid MAR, Azeem F, et al. Insights on Calcium-Dependent Protein Kinases (CPKs) Signaling for Abiotic Stress Tolerance in Plants. Int J Mol Sci. 2019;20(21):5298. doi: 10.3390/ijms20215298 31653073 PMC6862689

[8] Hamel L-P, Sheen J, Séguin A. Ancient signals: comparative genomics of green plant CDPKs. Trends Plant Sci. 2014;19(2):79–89. doi: 10.1016/j.tplants.2013.10.009 24342084 PMC3932502

[9] Boudsocq M, Sheen J. CDPKs in immune and stress signaling. Trends Plant Sci. 2013;18(1):30–40. doi: 10.1016/j.tplants.2012.08.008 22974587 PMC3534830

[10] Yip Delormel T, Boudsocq M. Properties and functions of calcium-dependent protein kinases and their relatives in *Arabidopsis thaliana*. New Phytol. 2019;224(2):585–604. doi: 10.1111/nph.16088 31369160

[11] Seybold H, Boudsocq M, Romeis T. CDPK Activation in PRR Signaling. Methods Mol Biol. 2017;1578:173–83. doi: 10.1007/978-1-4939-6859-6_14 28220424

[12] Liese A, Romeis T. Biochemical regulation of *in vivo* function of plant calcium-dependent protein kinases (CDPK). Biochim Biophys Acta. 2013;1833(7):1582–9. doi: 10.1016/j.bbamcr.2012.10.024 23123193

[13] da Cruz TI, Rocha DC, Lanna AC, Dedicova B, Vianello RP, Brondani C. Calcium-Dependent Protein Kinase 5 (OsCPK5) Overexpression in Upland Rice (*Oryza sativa* L.) under Water Deficit. Plants (Basel). 2023;12(22):3826. doi: 10.3390/plants12223826 38005723 PMC10674721

[14] Zhu X, Wang F, Li S, Feng Y, Yang J, Zhang N, et al. Calcium-Dependent Protein Kinase 28 Maintains Potato Photosynthesis and Its Tolerance under Water Deficiency and Osmotic Stress. Int J Mol Sci. 2022;23(15):8795. doi: 10.3390/ijms23158795 35955930 PMC9368905

[15] Wang B, Zhang Y, Bi Z, Liu Q, Xu T, Yu N, et al. Impaired Function of the Calcium-Dependent Protein Kinase, OsCPK12, Leads to Early Senescence in Rice (*Oryza sativa* L.). Front Plant Sci. 2019;10:52. doi: 10.3389/fpls.2019.00052 30778363 PMC6369234

[16] Hu J, Wang B, Yang T, Li N, Yang H, Yu Q, et al. A calcium-dependent protein kinase gene SpCPK33 from Solanum pennellii associated with increased cold tolerance in tomato. J Plant Physiol. 2022;279:153834. doi: 10.1016/j.jplph.2022.153834 36272175

[17] Yue J-Y, Jiao J-L, Wang W-W, Jie X-R, Wang H-Z. Silencing of the calcium-dependent protein kinase *TaCDPK27* improves wheat resistance to powdery mildew. BMC Plant Biol. 2023;23(1):134. doi: 10.1186/s12870-023-04140-y 36882703 PMC9993671

[18] Goher F, Bai X, Liu S, Pu L, Xi J, Lei J, et al. The Calcium-Dependent Protein Kinase TaCDPK7 Positively Regulates Wheat Resistance to *Puccinia striiformis* f. sp. *tritici*. Int J Mol Sci. 2024;25(2):1048. doi: 10.3390/ijms25021048 38256123 PMC10816280

[19] Li L, Yang M, Wei W, Zhao J, Yu X, Impaprasert R, et al. Characteristics of *Amorphophallus konjac* as indicated by its genome. Sci Rep. 2023;13(1):22684. doi: 10.1038/s41598-023-49963-9 38114626 PMC10730839

[20] Gao Y, Zhang Y, Feng C, Chu H, Feng C, Wang H, et al. A chromosome-level genome assembly of *Amorphophallus konjac* provides insights into konjac glucomannan biosynthesis. Comput Struct Biotechnol J. 2022;20:1002–11. doi: 10.1016/j.csbj.2022.02.009 35242290 PMC8860920

[21] Wei H, Yang M, Ke Y, Liu J, Chen Z, Zhao J, et al. Comparative physiological and transcriptomic profiles reveal regulatory mechanisms of soft rot disease resistance in *Amorphophallus* spp. Physiological and Molecular Plant Pathology. 2022;118:101807. doi: 10.1016/j.pmpp.2022.101807

[22] Newberg LA. Error statistics of hidden Markov model and hidden Boltzmann model results. BMC Bioinformatics. 2009;10:212. doi: 10.1186/1471-2105-10-212 19589158 PMC2722652

[23] Altschul SF, Gish W, Miller W, Myers EW, Lipman DJ. Basic local alignment search tool. J Mol Biol. 1990;215(3):403–10. doi: 10.1016/S0022-2836(05)80360-2 2231712

[24] Finn RD, Bateman A, Clements J, Coggill P, Eberhardt RY, Eddy SR, et al. Pfam: the protein families database. Nucleic Acids Res. 2014;42(Database issue):D222–30. doi: 10.1093/nar/gkt1223 24288371 PMC3965110

[25] Mistry J, Chuguransky S, Williams L, Qureshi M, Salazar GA, Sonnhammer ELL, et al. Pfam: The protein families database in 2021. Nucleic Acids Res. 2021;49(D1):D412–9. doi: 10.1093/nar/gkaa913 33125078 PMC7779014

[26] Bailey TL, Boden M, Buske FA, Frith M, Grant CE, Clementi L, et al. MEME SUITE: tools for motif discovery and searching. Nucleic Acids Res. 2009;37(Web Server issue):W202–8. doi: 10.1093/nar/gkp335 19458158 PMC2703892

[27] Chao J, Li Z, Sun Y, Aluko OO, Wu X, Wang Q, et al. MG2C: a user-friendly online tool for drawing genetic maps. Mol Hortic. 2021;1(1):16. doi: 10.1186/s43897-021-00020-x 37789491 PMC10514940

[28] Lescot M, Déhais P, Thijs G, Marchal K, Moreau Y, Van de Peer Y, et al. PlantCARE, a database of plant cis-acting regulatory elements and a portal to tools for in silico analysis of promoter sequences. Nucleic Acids Res. 2002;30(1):325–7. doi: 10.1093/nar/30.1.325 11752327 PMC99092

[29] Katoh K, Standley DM. MAFFT multiple sequence alignment software version 7: improvements in performance and usability. Mol Biol Evol. 2013;30(4):772–80. doi: 10.1093/molbev/mst010 23329690 PMC3603318

[30] Kumar S, Nei M, Dudley J, Tamura K. MEGA: a biologist-centric software for evolutionary analysis of DNA and protein sequences. Brief Bioinform. 2008;9(4):299–306. doi: 10.1093/bib/bbn017 18417537 PMC2562624

[31] Letunic I, Bork P. Interactive Tree of Life (iTOL) v6: recent updates to the phylogenetic tree display and annotation tool. Nucleic Acids Res. 2024;52(W1):W78–82. doi: 10.1093/nar/gkae268 38613393 PMC11223838

[32] Waterhouse AM, Procter JB, Martin DMA, Clamp M, Barton GJ. Jalview Version 2--a multiple sequence alignment editor and analysis workbench. Bioinformatics. 2009;25(9):1189–91. doi: 10.1093/bioinformatics/btp033 19151095 PMC2672624

[33] Hu B, Jin J, Guo A-Y, Zhang H, Luo J, Gao G, et al. GSDS 2.0: an upgraded gene feature visualization server. Bioinformatics. 2015;31(8):1296–7. doi: 10.1093/bioinformatics/btu817 25504850 PMC4393523

[34] Wang D, Zhang Y, Zhang Z, Zhu J, Yu J. KaKs_Calculator 2.0: a toolkit incorporating gamma-series methods and sliding window strategies. Genomics Proteomics Bioinformatics. 2010;8(1):77–80. doi: 10.1016/S1672-0229(10)60008-3 20451164 PMC5054116

[35] Wang Y, Tang H, Debarry JD, Tan X, Li J, Wang X, et al. MCScanX: a toolkit for detection and evolutionary analysis of gene synteny and collinearity. Nucleic Acids Res. 2012;40(7):e49. doi: 10.1093/nar/gkr1293 22217600 PMC3326336

[36] Livak KJ, Schmittgen TD. Analysis of relative gene expression data using real-time quantitative PCR and the 2^-ΔΔCT^ Method. Methods. 2001;25(4):402–8. doi: 10.1006/meth.2001.1262 11846609

[37] Cheng S-H, Willmann MR, Chen H-C, Sheen J. Calcium signaling through protein kinases. The Arabidopsis calcium-dependent protein kinase gene family. Plant Physiol. 2002;129(2):469–85. doi: 10.1104/pp.005645 12068094 PMC1540234

[38] Asano T, Tanaka N, Yang G, Hayashi N, Komatsu S. Genome-wide identification of the rice calcium-dependent protein kinase and its closely related kinase gene families: comprehensive analysis of the CDPKs gene family in rice. Plant Cell Physiol. 2005;46(2):356–66. doi: 10.1093/pcp/pci035 15695435

[39] Ray S, Agarwal P, Arora R, Kapoor S, Tyagi AK. Expression analysis of calcium-dependent protein kinase gene family during reproductive development and abiotic stress conditions in rice (*Oryza sativa* L. ssp. *indica*). Mol Genet Genomics. 2007;278(5):493–505. doi: 10.1007/s00438-007-0267-4 17636330

[40] Liu X, Zeeshan Ul Haq M, Yu J, Liu Y, Yang H, Cui H, et al. Identification of the *CDPK* gene family in patchouli and functional analysis in response to continuous cropping stress. Front Plant Sci. 2023;14:1300073. doi: 10.3389/fpls.2023.1300073 38078089 PMC10702526

[41] Zhao P, Liu Y, Kong W, Ji J, Cai T, Guo Z. Genome-Wide Identification and Characterization of Calcium-Dependent Protein Kinase (CDPK) and CDPK-Related Kinase (CRK) Gene Families in *Medicago truncatula*. Int J Mol Sci. 2021;22(3):1044. doi: 10.3390/ijms22031044 33494310 PMC7864493

[42] Zhang B, Song Y, Zhang X, Wang Q, Li X, He C, et al. Identification and expression assay of calcium-dependent protein kinase family genes in *Hevea brasiliensis* and determination of *HbCDPK5* functions in disease resistance. Tree Physiol. 2022;42(5):1070–83. doi: 10.1093/treephys/tpab156 35022787

[43] Nuruzzaman M, Manimekalai R, Sharoni AM, Satoh K, Kondoh H, Ooka H, et al. Genome-wide analysis of NAC transcription factor family in rice. Gene. 2010;465(1–2):30–44. doi: 10.1016/j.gene.2010.06.008 20600702

[44] Li Y, Zhang H, Liang S, Chen X, Liu J, Zhang Y, et al. Identification of the *CDPK* Gene Family in *Solanum habrochaites* and Its Function Analysis under Stress. Int J Mol Sci. 2022;23(8):4227. doi: 10.3390/ijms23084227 35457042 PMC9031491

[45] Fedorowicz-Strońska O, Koczyk G, Kaczmarek M, Krajewski P, Sadowski J. Genome-wide identification, characterisation and expression profiles of calcium-dependent protein kinase genes in barley (*Hordeum vulgare* L.). J Appl Genet. 2017;58(1):11–22. doi: 10.1007/s13353-016-0357-2 27447459 PMC5243917

[46] Vision TJ, Brown DG, Tanksley SD. The origins of genomic duplications in *Arabidopsis*. Science. 2000;290(5499):2114–7. doi: 10.1126/science.290.5499.2114 11118139

[47] Wu P, Wang W, Duan W, Li Y, Hou X. Comprehensive Analysis of the CDPK-SnRK Superfamily Genes in Chinese Cabbage and Its Evolutionary Implications in Plants. Front Plant Sci. 2017;8:162. doi: 10.3389/fpls.2017.00162 28239387 PMC5301275

[48] Fan S, Yang S, Li G, Wan S. Genome-Wide Identification and Characterization of CDPK Gene Family in Cultivated Peanut (*Arachis hypogaea* L.) Reveal Their Potential Roles in Response to Ca Deficiency. Cells. 2023;12(23):2676. doi: 10.3390/cells12232676 38067104 PMC10705679

[49] Myers C, Romanowsky SM, Barron YD, Garg S, Azuse CL, Curran A, et al. Calcium-dependent protein kinases regulate polarized tip growth in pollen tubes. Plant J. 2009;59(4):528–39. doi: 10.1111/j.1365-313X.2009.03894.x 19392698

[50] Ding Y, Yang H, Wu S, Fu D, Li M, Gong Z, et al. CPK28-NLP7 module integrates cold-induced Ca^2+^ signals and transcriptional reprogramming in *Arabidopsis*. Sci Adv. 2022;8(26):eabn7901. doi: 10.1126/sciadv.abn7901 35767615 PMC9242591

[51] Yue J-Y, Jiao J-L, Wang W-W, Wang H-Z. The Calcium-Dependent Protein Kinase TaCDPK27 Positively Regulates Salt Tolerance in Wheat. Int J Mol Sci. 2022;23(13):7341. doi: 10.3390/ijms23137341 35806346 PMC9266408

[52] Grossi CEM, Santin F, Quintana SA, Fantino E, Ulloa RM. Calcium-dependent protein kinase 2 plays a positive role in the salt stress response in potato. Plant Cell Rep. 2022;41(3):535–48. doi: 10.1007/s00299-021-02676-7 33651205

[53] Cheng H, Pan G, Zhou N, Zhai Z, Yang L, Zhu H, et al. Calcium-dependent Protein Kinase 5 (CPK5) positively modulates drought tolerance through phosphorylating ABA-Responsive Element Binding Factors in oilseed rape (*Brassica napus* L.). Plant Sci. 2022;315:111125. doi: 10.1016/j.plantsci.2021.111125 35067297

[54] Bundó M, Coca M. Calcium-dependent protein kinase OsCPK10 mediates both drought tolerance and blast disease resistance in rice plants. J Exp Bot. 2017;68(11):2963–75. doi: 10.1093/jxb/erx145 28472292 PMC5853374

[55] Godwin J, Farrona S. Plant Epigenetic Stress Memory Induced by Drought: A Physiological and Molecular Perspective. Methods Mol Biol. 2020;2093:243–59. doi: 10.1007/978-1-0716-0179-2_17 32088901

[56] Sato H, Mizoi J, Shinozaki K, Yamaguchi-Shinozaki K. Complex plant responses to drought and heat stress under climate change. Plant J. 2024;117(6):1873–92. doi: 10.1111/tpj.16612 38168757

