# Correction: Identification of the *AKCDPK* gene family and *AkCDPK15* functional analysis under drought and salt stress

**DOI:** 10.1371/journal.pone.0332416

**Published:** 2025-09-12

**Authors:** Penghua Gao, Ying Zou, Min Yang, Lifang Li, Ying Qi, Jianwei Guo, Yongteng Zhao, Jiani Liu, Jianrong Zhao, Feiyan Huang, Lei Yu

There is an error in the affiliation of the authors. The correct affiliation is: College of Agronomy, Yunnan Urban Agricultural Engineering and Technological Research Center, Yunnan Provincial Key Laboratory of Konjac Biology, Kunming University, Kunming, China.
