# Supplementary figures and images for: Identification of the AKCDPK gene family and AkCDPK15 functional analysis under drought and salt stress
Source: PLoS One. 2025 Jun 11;20(6):e0325453. doi: 10.1371/journal.pone.0325453 (PMC12157117; doi:10.1371/journal.pone.0325453)

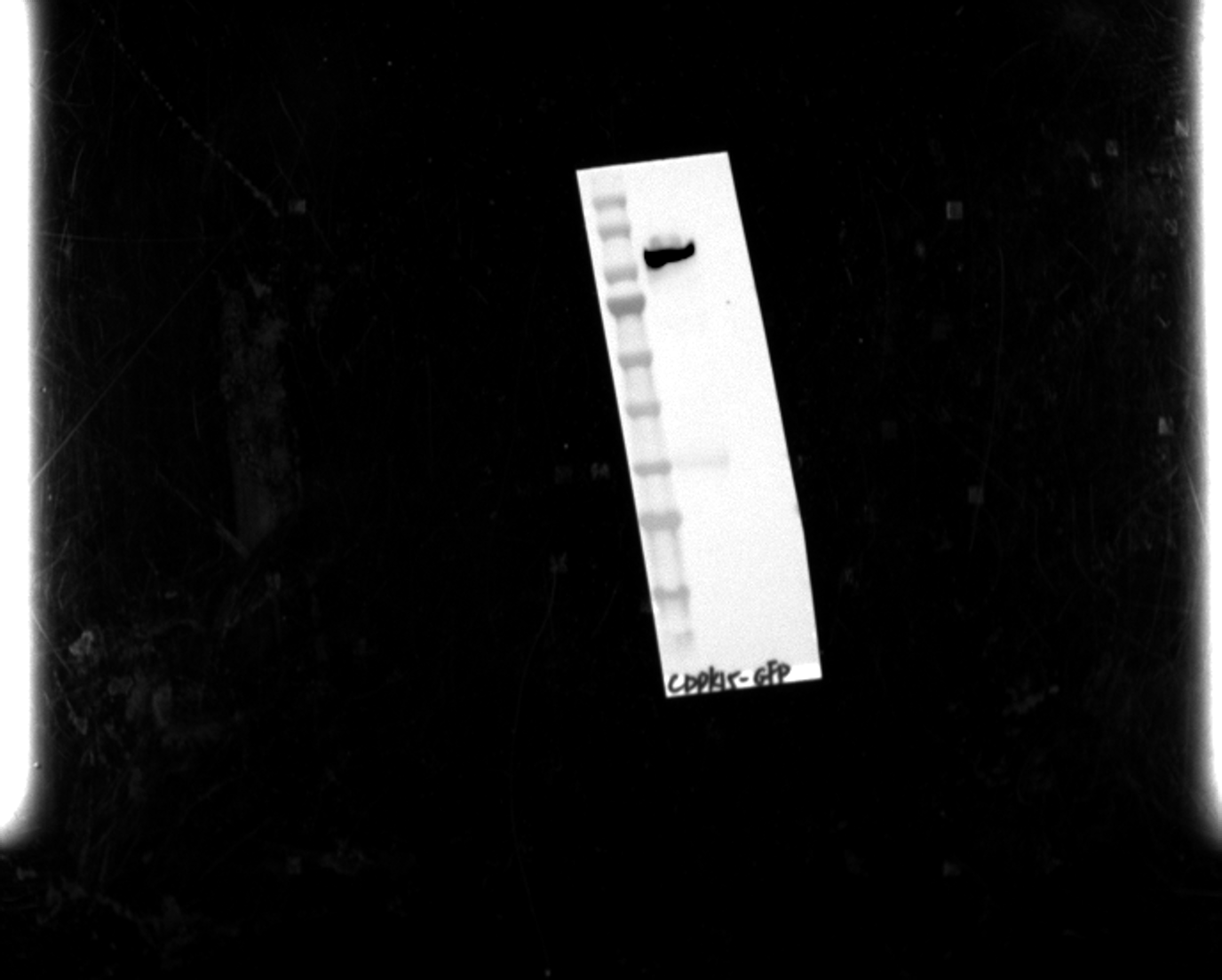

Supplement: S1 Fig — (TIF) [file pone.0325453.s008.tif]

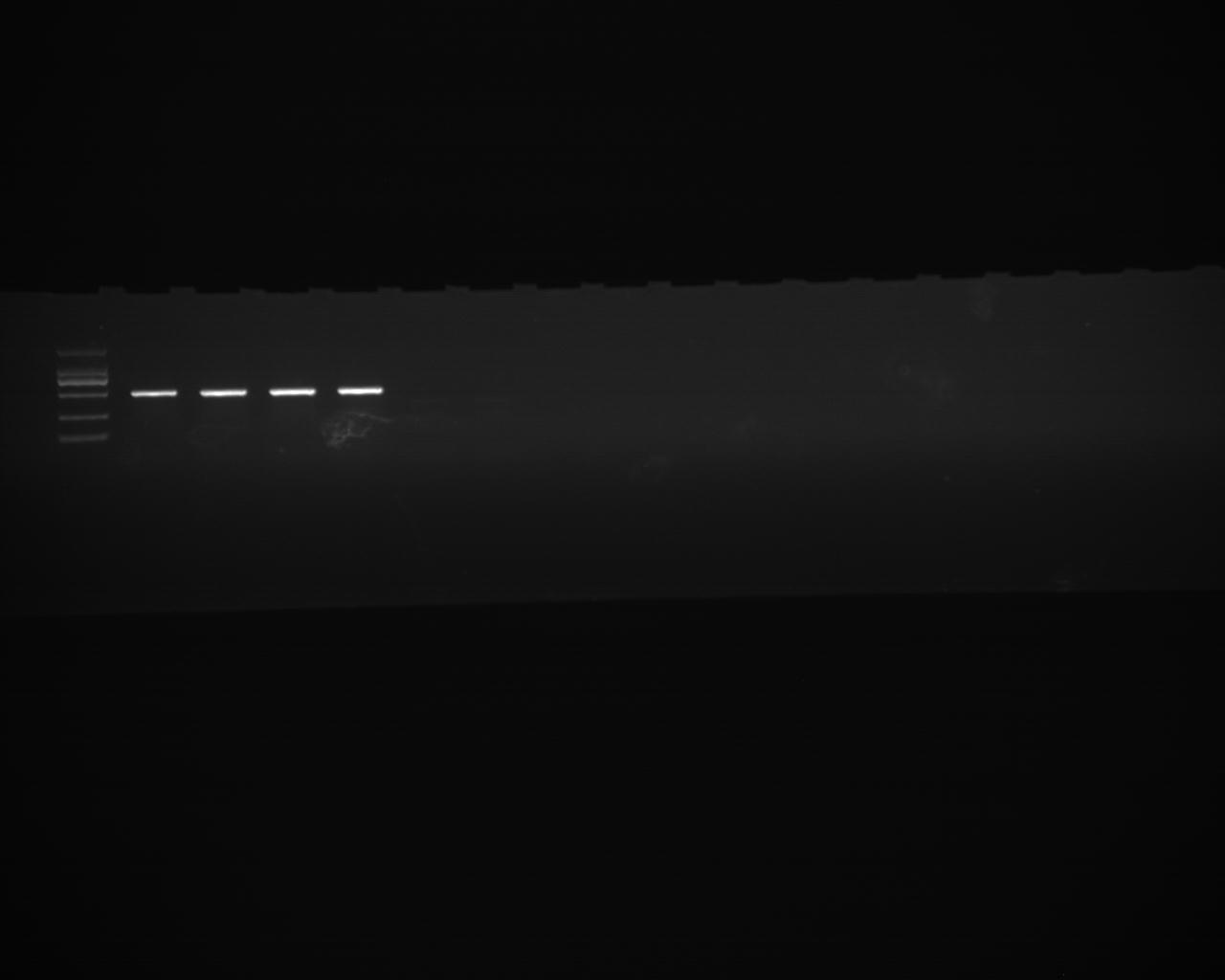

Supplement: S2 Fig — (TIF) [file pone.0325453.s009.tif]

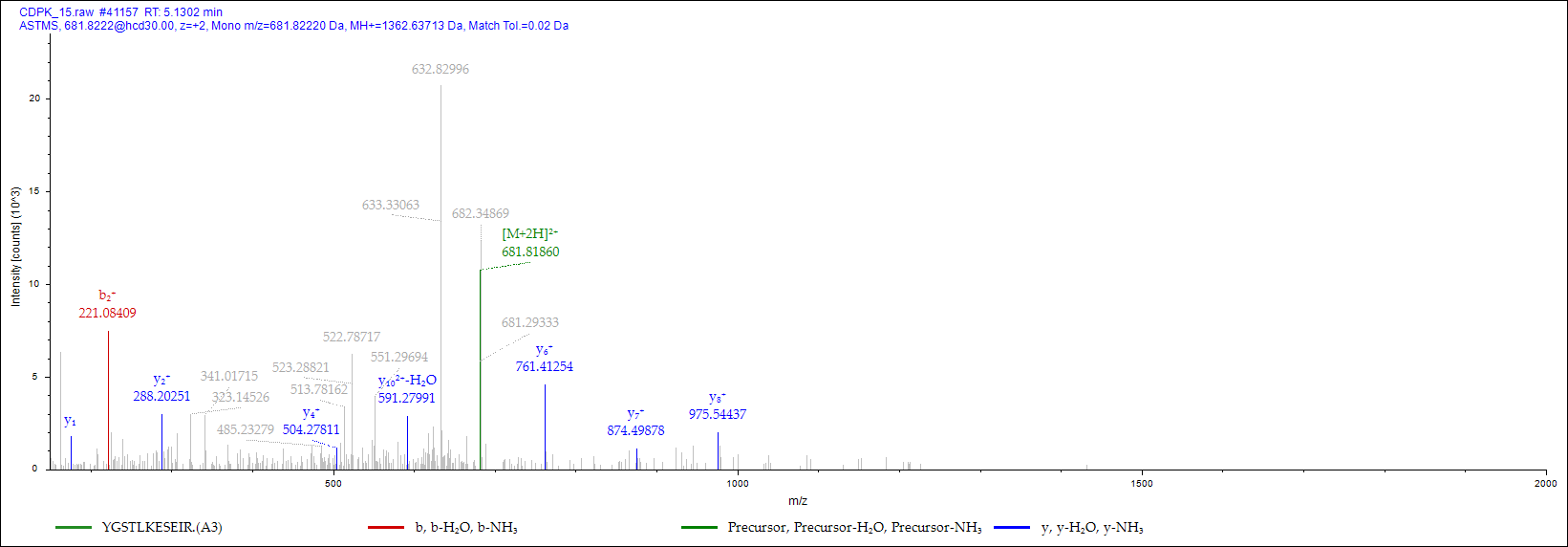

Supplement: S3 Fig — (TIF) [file pone.0325453.s010.tif]
